# Semantic Richness Effects in Spoken Word Recognition: A Lexical Decision and Semantic Categorization Megastudy

**DOI:** 10.3389/fpsyg.2016.00976

**Published:** 2016-06-28

**Authors:** Winston D. Goh, Melvin J. Yap, Mabel C. Lau, Melvin M. R. Ng, Luuan-Chin Tan

**Affiliations:** Department of Psychology, National University of SingaporeSingapore, Singapore

**Keywords:** semantic richness, megastudy, spoken word recognition, lexical decision, semantic categorization

## Abstract

A large number of studies have demonstrated that semantic richness dimensions [e.g., number of features, semantic neighborhood density, semantic diversity , concreteness, emotional valence] influence word recognition processes. Some of these richness effects appear to be task-general, while others have been found to vary across tasks. Importantly, almost all of these findings have been found in the visual word recognition literature. To address this gap, we examined the extent to which these semantic richness effects are also found in spoken word recognition, using a megastudy approach that allows for an examination of the relative contribution of the various semantic properties to performance in two tasks: lexical decision, and semantic categorization. The results show that concreteness, valence, and number of features accounted for unique variance in latencies across both tasks in a similar direction—faster responses for spoken words that were concrete, emotionally valenced, and with a high number of features—while arousal, semantic neighborhood density, and semantic diversity did not influence latencies. Implications for spoken word recognition processes are discussed.

## Introduction

The goal of speech perception is to understand the meaning of spoken words and sentences. However, much of the research in the field of spoken word recognition has focused on the effects of lexical variables such as word frequency and structural variables such as word-form similarity. Frequency effects (i.e., common words such as *cat* are recognized faster than uncommon words such as *wag*) have been well-established. Word-form similarity between the target word and other words in the mental lexicon have also been shown to influence recognition latencies. One measure of structural similarity is phonological neighborhood density (*N*-metric: Luce and Pisoni, 1998), which indexes the number of words that differ from the target word by a single phoneme. Words with dense neighborhoods (*cat* has many neighbors such as *hat, cut, at, catty*) are recognized more slowly than words with sparse neighborhoods (*wag* has fewer neighbors such as *bag, wan*; e.g., Luce and Pisoni, 1998; Ziegler et al., 2003; Goh et al., 2009). Results from studies using other metrics of word-form similarity such as the clustering coefficient (*C*-metric: Watts and Strogatz, 1998) and neighborhood spread (*P*-metric: Andrews, 1997) all converge on the general finding that lexical competition between similar sounding words slow down spoken word recognition (Vitevitch, 2007; Chan and Vitevitch, 2009).

More recent studies continue to examine structural influences, investigating phonological similarity effects beyond the single phoneme difference, such as phonological Levenshtein distance (PLD20: Suárez et al., 2011), and the global phonological network characteristics of the mental lexicon (Siew and Vitevitch, 2015). The pattern of results again suggest robust effects of lexical competition—the more distinct the word-form, the faster the word gets recognized. The focus on lexical and structural characteristics in spoken word recognition research is perhaps unsurprising when one considers the fact that extracting and identifying a word or series of words from a continuous acoustic signal is a unique challenge for speech perception where, unlike reading printed words, there are no clear cut boundaries that indicate where one word ends and another begins (see Goldinger et al., 1996).

### Semantic richness effects in word recognition

However, when we consider what the ultimate goal of listening as well as reading is, it is clear that there is a common aim for both modalities—the semantics of the message. Compared to spoken word recognition, the field of visual word recognition is far more advanced in examining semantic influences across dimensions as well as tasks.

Several semantic dimensions have been found to influence visual word recognition to some degree. These dimensions include number of features (NoF)—the number of attributes that people can list for each concept (McRae et al., 2005), concreteness—the extent to which words evoke sensory and motor experiences (Brysbaert et al., 2014), semantic neighborhood density (SND)—the extent to which words co-occur with other words in the language (Shaoul and Westbury, 2010), semantic diversity (SD)—a word's variability in its contextual usage, an estimate of semantic ambiguity (Hoffman et al., 2013), and emotional valence and arousal (Russell, 1980)—the emotional characteristics of words, such as whether they are positive or negative emotion words (valence) and the extent to which emotional words elicit a physiological reaction (arousal; Bradley and Lang, 1999; Warriner et al., 2013). Specifically, the more robust findings indicate that printed words are recognized faster when they are associated with referents with more features (Pexman et al., 2002), when they reside in denser semantic neighborhoods (Buchanan et al., 2001), and when they are concrete (Schwanenflugel, 1991).

The effects of valence and arousal are more mixed (Kuperman et al., 2014). For example, there is some debate on whether the relation between valence and word recognition is linear and monotonic (i.e., faster recognition for positive words; Kuperman et al., 2014) or is represented by a non-monotonic, inverted U (i.e., faster recognition for valenced, compared to neutral, words; Kousta et al., 2009). Additionally, it is unclear if valence and arousal produce additive (Kuperman et al., 2014) or interactive (Larsen et al., 2006) effects. Specifically, Larsen et al. (2006) reported that valence effects were larger for low-arousal than for high-arousal words in lexical decision, but Kuperman et al. (2014) found no evidence for such an interaction in their analysis of over 12,000 words.

In general, these findings converge on the idea that words with richer semantic representations are recognized faster. Pexman (2012) has suggested that these semantic richness effects contribute to word recognition processes via cascaded interactive activation mechanisms that allow feedback from semantic to lexical representations (see Yap et al., 2015).

Turning to task factors, the evidence suggests that the magnitude of semantic richness effects as well as the relative contributions of each semantic dimension differs across tasks. In general, the magnitude of richness effects is greater for semantic categorization tasks (e.g., deciding whether a word is abstract or concrete) compared to lexical decision (categorizing the target stimulus as a word or nonword). The explanation is that tasks requiring lexical judgments emphasize the word's form, and hence non-semantic variables explain more of the unique variance, whereas tasks requiring meaningful judgments require semantic analysis, which then tap more on the semantic properties (Pexman et al., 2008). Furthermore, some of the semantic dimensions influence response latencies across tasks to varying degrees, while others have been found to influence latencies in some tasks but not others. For example, SND affects lexical decision but not semantic classification, whereas NoF affects both but more strongly for semantic classification (Pexman et al., 2008; Yap et al., 2011). One explanation that has been advanced is that close semantic neighbors facilitate semantic classification, whereas distant neighbors inhibit responses, leading to a trade-off in the net effect of SND (Mirman and Magnuson, 2006). The effect of NoF across both tasks reflect greater feedback activation levels from the semantic representations to the orthographic representations in supporting faster lexical decisions, and faster semantic activation to support more rapid semantic classification. These patterns of results suggest that the influence of semantic properties is multifaceted and involves both task-general and task-specific processes.

### The present study

While there have been rapid advances in the investigation of semantic influences on visual word recognition, only a couple of studies have thus far examined richness effects in spoken word recognition. Tyler et al. (2000) observed that concrete words (high imageability) elicited faster responses than abstract words (low imageability) in auditory lexical decision and speeded repetition. Sajin and Connine (2014) found that the NoF effect observed in visual word recognition was replicated with spoken words—words with high NoF were recognized faster than those with low NoF in auditory lexical decision. Both studies further found that the concreteness and NoF effects were more evident when there was greater competition among potential words, either via cohort sizes, onset competitors, or sub-optimal listening conditions.

The present study aims to address the gap in the spoken word recognition field with respect to the *relative* contributions of semantic properties to auditory word processing. Tyler et al. (2000) only examined concreteness, while Sajin and Connine (2014) only examined NoF. Pexman (2012) has suggested that the different semantic indices tap unique dimensions, and given the variability in the magnitude and nature of the influence among the semantic dimensions that has been found in visual word recognition, it is important to determine the extent to which the richness effects also occur in spoken word recognition and if there are any differences compared to visual word recognition. While the goal of listening and reading may ultimately be the same, the work on lexical processing in both fields have shown that some of the effects do not generalize across modalities.

For example, dense phonological neighborhoods consistently slow down processing of spoken words, whereas orthographic neighborhood effects are more mixed in visual word recognition (Andrews, 1997). The interaction between word frequency and phonological neighborhood density shows that density effects are larger for high-frequency, compared to low-frequency, words in spoken word recognition (Luce and Pisoni, 1998; Goh et al., 2009). However, the opposite pattern, i.e., smaller density effects for high-frequency words is observed in visual word recognition (Andrews, 1989, 1992). This means that in spoken word recognition, the advantage of high frequency words is attenuated when there is more word-form competition, suggesting that the recognition process in speech may focus more on resolving phonological similarities first (Luce and Pisoni, 1998; Goh et al., 2009). These dissociations between the patterns in visual and spoken word recognition point to the importance of investigating modality-specific and modality-general influences for semantic richness.

The megastudy approach (Balota et al., 2004) was adopted as it is more appropriate compared to factorial designs for examining the relative contributions of each of the semantic dimensions. Stimuli properties need not be matched or manipulated, and the unique contributions of semantic richness factors that explain the variance in response latencies above and beyond the variance explained by structural and lexical variables can be examined. We also examined richness effects across two different tasks, lexical decision, and semantic categorization, given the previous findings demonstrating task-specific and task-general effects.

## Method

### Participants

Eighty students from the National University of Singapore (NUS) were paid SGD10 for participation. Forty did the lexical decision task (LDT) while 40 did the semantic categorization task (SCT). All were native speakers of English and had no speech or hearing disorder at the time of testing. Participation occurred with informed consent and protocols were approved by the NUS Institutional Review Board.

### Materials

The words of interest were the 514 concrete nouns from McRae et al. (2005). A trained linguist who was a female native speaker of Singapore English was recruited for recording the tokens in 16-bit mono, 44.1 kHz.wav sound files. These files were then digitally normalized to 70 dB to ensure that all tokens had the same overall root-mean-square amplitudes. The tokens were then presented to 20 participants from the same population sample, but who did not take part in the main study, to check for correct identification of the target words. Tokens that did not achieve at least 80% correct identification were re-recorded and re-tested. Tokens were also checked for homophone responses (e.g., *flea/flee, hare/hair*). These issues led to 46 words eventually dropped from the set after the second round of testing.

The two tasks used different distracters. Specifically, abstract words were the distracters in the SCT while nonwords were the distracters in the LDT. For the SCT, 530 abstract nouns from Pexman et al. (2008) were then recorded by the same speaker and checked for identifiability and if they were homophones. An eventual 468 abstract words were chosen that were matched as closely as possible to the 468 concrete words of interest on log subtitle word frequency, phonological neighborhood density, PLD20, number of phonemes, syllables, morphemes, and identification rates using the Match program (Van Casteren and Davis, 2007). For the LDT, 468 nonwords were also recorded by the speaker. The nonwords were generated using Wuggy (Keuleers and Brysbaert, 2010) and checked that they did not include homophones for the spoken tokens.

The average identification scores for all word tokens was 93.69% (*SD* = 7.74). The predictor variables for the concrete nouns were divided into two clusters representing lexical and semantic variables; Table 1 lists descriptive statistics of all predictor and dependent variables used in the analyses.

**Table 1:** **Means and standard deviations for predictor variables and dependent measures (***N*** = 357)**.

**Variable**	***M***	***SD***
Word duration (ms)	630.44	116.38
Log subtitle word frequency	2.61	0.58
Uniqueness point	3.51	1.04
Phonological neighborhood density	8.00	9.52
Phonological Levenshtein distance	2.02	0.99
No. of phonemes	4.75	1.64
No. of syllables	1.76	0.79
No. of morphemes	1.22	0.47
Concreteness	4.85	0.14
Valence	5.59	0.92
Arousal	3.85	0.91
Number of features	12.39	3.26
Semantic neighborhood density	0.53	0.10
Semantic diversity	1.46	0.25
RT LDT (ms)	918.82	99.05
Z-RT LDT	−0.43	0.35
Accuracy LDT	0.94	0.07
RT SCT (ms)	997.10	109.71
Z-RT SCT	−0.27	0.37
Accuracy SCT	0.91	0.07

#### Lexical variables

These included word duration, measured from the onset of the token's waveform to the offset, which corresponded to the duration of the edited soundfiles, log subtitle word frequency (Brysbaert and New, 2009), uniqueness point (i.e., the point at which a word diverges from all other words in the lexicon; Luce, 1986), phonological Levenshtein distance (Yap and Balota, 2009), phonological neighborhood density, number of phonemes, number of syllables, and number of morphemes (all taken from the English Lexicon Project, Balota et al., 2007). Brysbaert and New's (2009) frequency norms are based on a corpus of television and film subtitles and have been shown to predict word processing times better than other available measures. More importantly, they are more likely to provide a good approximation of exposure to spoken language in the real world.

#### Semantic variables

Concreteness ratings were taken from Brysbaert et al. (2014), emotional valence, and arousal ratings were obtained from Warriner et al. (2013), NoF was from McRae et al. (2005), SND was based on the average radius of co-occurrence values (Shaoul and Westbury, 2010), and SD from Hoffman et al. (2013).

### Procedure

Participants were tested in groups of five or fewer on individual computers running E-prime 1.2 and PST Serial Response Boxes (Schneider et al., 2002), with the left- and right-most buttons labeled nonword and word, respectively, for the LDT, and abstract and concrete, respectively, for the SCT. Stimuli were binaurally played through beyerdynamic DT150 headphones at ~70 dB SPL. Participants were instructed to classify as quickly and as accurately whether each word was a word or nonword for the LDT, and whether each word was abstract or concrete for the SCT.

A number of researchers have suggested that there are differences in semantic effects between tasks requiring decisions based on narrow (e.g., bird/non-bird) vs. broad (e.g., living/non-living) dichotomies. For example, Pexman et al. (2003) examined the influence of NoF in three different semantic decision tasks. They found that effects were largest in abstract/concrete SCTs, with smaller effects for living/non-living and bird/non-bird SCTs. For the narrower SCTs, participants may focus only on the diagnostic dimension for those categories. It has been recommended that researchers avoid over-specific categories (e.g., Jared and Seidenberg, 1991), and we chose the broad abstract/concrete distinction to maximize the number of items that can be presented under common task demands (Pexman et al., 2016).

Response times (RT) were measured from stimulus onset to the button press. The first 20 trials were for practice, using tokens unrelated to the study, followed by 936 experimental trials randomized for each participant across 6 blocks of 156 trials each. The inter-trial interval was 500 ms, with a short break after each block.

## Results

Following Pexman et al. (2008), we first excluded trials associated with items that yielded an accuracy rate lower than 70% in either the LDT or SCT, before excluding incorrect responses [mean accuracy was 89% in the LDT (*SD* = 4.9), and 92% in the SCT (*SD* = 4.0)]. Next, responses which were faster than 200 ms or slower than 3000 ms were excluded (0.5% in the LDT; 0.1% in the SCT), before removing trials that were more than 2.5 *SD*s away from each participant's mean (2.4% in the LDT; 2.6% in the SCT). We then standardized raw RTs using a *z*-score transformation; *z*-score transformed RTs have the advantage of minimizing the influence of a participant's processing speed and variability (Faust et al., 1999).

Of the items remaining, this left 357 concrete nouns which had values on the lexical and semantic variables examined. Table 2 shows the correlation matrix for all variables. The semantic variables were either uncorrelated, or weakly to modestly correlated with each other, consistent with earlier suggestions that they tap distinct constructs (Pexman et al., 2008). In addition, because of the very high correlations (|*r*|s between 0.66 and 0.89) between the length and neighborhood density measures, principal components analysis (PCA) was used to reduce phonological neighborhood density, phonological Levenshtein distance, number of phonemes, and number of syllables to a single component; varimax rotation with Kaiser normalization was used. The component accounted for 83% of the variance and appears to capture the structural properties of words, with higher values denoting greater phonological distinctiveness (see Table 3 for component loadings).

**Table 2:** **Correlation matrix for all variables (***N*** = 357)**.

	**1**	**2**	**3**	**4**	**5**	**6**	**7**	**8**	**9**	**10**	**11**	**12**	**13**	**14**	**15**	**16**	**17**	**18**	**19**	**20**
1. Duration	−	−0.34^***^	−0.27^***^	−0.46^***^	0.62^***^	0.66^***^	0.48^***^	0.43^***^	0.11^*^	−0.03	0.09^†^	−0.01	−0.28^***^	−0.21^***^	0.51^***^	0.53^***^	0.08	0.49^***^	0.51^***^	0.07
2. Word frequency		−	−0.11^*^	0.46^***^	−0.48^***^	−0.45^***^	−0.40^***^	−0.28^***^	0.08	0.08	0.05	0.27^***^	0.73^***^	0.45^***^	−0.33^***^	−0.34^***^	0.16^**^	−0.27^***^	−0.28^***^	0.10^*^
3. Uniqueness point			−	−0.29^***^	0.32^***^	0.49^***^	0.32^***^	0.35^***^	−0.05	−0.06	0.12^*^	0.03	−0.10^†^	−0.10^†^	0.11^*^	0.12^*^	0.15^**^	0.12^*^	0.12^*^	0.02
4. Phonological N				−	−0.68^***^	−0.71^***^	−0.66^***^	−0.34^***^	−0.03	0.04	−0.10^†^	0.05	0.39^***^	0.34^***^	−0.08	−0.09^†^	−0.14^**^	−0.08	−0.09^†^	−0.11^*^
5. Phonological LD					−	0.89^***^	0.81^***^	0.54^***^	0.04	0.02	0.11^*^	0.00	−0.42^***^	−0.34^***^	0.14^**^	0.16^**^	0.19^***^	0.10^†^	0.11^*^	0.21^***^
6. No. of phonemes						−	0.85^***^	0.51^***^	0.02	0.04	0.11^*^	0.00	−0.37^***^	−0.32^***^	0.16^**^	0.18^**^	0.20^***^	0.16^**^	0.17^**^	0.13^*^
7. No. of syllables							−	0.41^***^	−0.01	0.06	0.11^*^	0.00	−0.28^***^	−0.26^***^	0.02	0.04	0.23^***^	0.07	0.07	0.12^*^
8. No. of morphemes								−	0.01	−0.06	0.08	0.00	−0.34^***^	−0.26^***^	0.26^***^	0.28^***^	0.05	0.23^***^	0.23^***^	0.09^†^
9. Concreteness									−	0.06	−0.05	0.22^***^	0.01	−0.01	−0.10^†^	−0.11^*^	0.12^*^	−0.22^***^	−0.20^***^	0.31^***^
10. Valence										−	−0.27^***^	0.06	0.09^†^	−0.03	−0.19^***^	−0.19^***^	0.06	−0.19^***^	−0.18^**^	0.12^*^
11. Arousal											−	0.06	0.14^**^	−0.04	0.02	0.02	0.05	0.04	0.04	−0.05
12. No. of features												−	0.13^*^	0.04	−0.19^***^	−0.19^***^	0.18^**^	−0.22^***^	−0.22^***^	0.26^***^
13. Semantic N													−	0.41^***^	−0.27^***^	−0.28^***^	0.12^*^	−0.19^***^	−0.19^***^	0.01
14. Sem diversity														−	−0.13^*^	−0.14^†^	−0.02	−0.11^*^	−0.12^*^	−0.03
15. RT LDT															−	1.00^***^	−0.51^***^	0.84^***^	0.86^***^	−0.36^***^
16. ZRT LDT																−	−0.49^***^	0.85^***^	0.87^***^	−0.35^***^
17. Accuracy LDT																	−	−0.41^***^	−0.42^***^	0.46^***^
18. RT SCT																		−	0.99^***^	−0.43^***^
19. ZRT SCT																			−	−0.44^***^
20. Accuracy SCT																				−

† *p < 0.10*,

* *p < 0.05*,

** *p < 0.01*,

*** *p < 0.001*.

**Table 3:** **Principal component loadings**.

**Predictors**	**Loading**
1. Phonological neighborhood density	−0.83
2. Phonological Levenshtein distance	0.94
3. Number of phonemes	0.95
4. Number of syllables	0.92

Hierarchical regression analyses were conducted with *z*-score transformed RT as the criterion. The lexical control variables were entered as predictor variables in the first step, the semantic richness variables were entered in the second step, the quadratic valence term in Step 3, and the interaction terms between valence and arousal in Step 4. Table 4 lists the results of the regression analyses. In general, multicollinearity was not an issue; the tolerance values of the lexical and semantic predictors ranged between 0.42 and 0.92.

**Table 4:** **LDT and SCT standardized regression coefficients for item-level hierarchical regression analyses**.

**Variable**	**LDT**	**SCT**
**STEP 1: LEXICAL VARIABLES (CONTROL)**		
Word duration	0.68^***^	0.65^***^
Log subtitle word frequency	−0.34^***^	−0.27^***^
Uniqueness point	0.07	0.08^†^
Structural principal component	−0.55^***^	−0.48^***^
No. of morphemes	0.14^**^	0.08
Adjusted *R*^2^	0.445^***^	0.367^***^
**STEP 2: SEMANTIC RICHNESS VARIABLES**		
Concreteness	−0.12^**^	−0.21^***^
Valence	−0.11^**^	−0.12^**^
Arousal	−0.01	−0.02
Number of features	−0.08^*^	−0.12^**^
Semantic neighborhood density	−0.03	0.05
Semantic diversity	0.00	−0.04
Δ*R*^2^	0.030^***^	0.075^***^
**STEP 3: QUADRATIC VALENCE**		
Valence^2^	−0.14^**^	−0.11^*^
Δ*R*^2^	0.011^**^	0.007^*^
**STEP 4: VALENCE** × **AROUSAL**		
Valence × Arousal	0.04	0.11
Valence^2^ × Arousal	0.02	0.08
Δ*R*^2^	0.000	0.001

*Coefficients reflect those entered in the corresponding step*.

† *p < 0.10*,

* *p < 0.05*,

** *p < 0.01*,

*** *p < 0.001*.

For the LDT, the lexical control variables collectively accounted for 44.5% of the variance in RT, *F*_(5, 351)_ = 58.04, *p* < 0.001. There were significant positive relationships between RT and token duration and number of morphemes. Words that had longer tokens and more morphemes were associated with slower RTs. There were also significant negative relationships between RT and frequency and the structural principal component (PC). Higher frequency and more phonologically distinct (i.e., less confusable) words were responded to faster.

Semantic richness variables collectively accounted for an additional 3% of unique variance in RT, above and beyond the variance already accounted for by the lexical variables, *F*_change(6, 345)_ = 4.31, *p* < 0.001. There were significant negative relationships between RT and concreteness, valence, and NoF. More concrete words, positively valenced words, and words with a higher NoF had faster RTs. There was no significant relationship between RT and arousal, SND, and SD.

Turning to non-linear effects, the quadratic valence term accounted for an additional 1.1% of variance, *F*_change(1, 344)_ = 8.93, *p* = 0.003. The negative regression coefficient indicates an inverted U-shaped relationship, in which very negative and very positive words were responded to faster than neutral words. Finally, arousal did not interact with either linear or quadratic valence, *F*_change_ < 1.

Turning to SCT, the lexical control variables collectively accounted for 36.7% of the variance in RT, *F*_(5, 351)_ = 42.26, *p* < 0.001. There was a significant relationship between RT and word duration; words that had longer tokens were associated with slower RTs. There were also significant negative relationships between RT and frequency and the structural PC. Higher frequency and more phonologically distinct words were responded to faster.

Semantic richness variables collectively accounted for an additional 7.5% of unique variance in RT, above and beyond the variance already accounted for by the lexical variables, *F*_change(6, 345)_ = 8.88, *p* < 0.001. There were significant negative relationships between RT and concreteness, valence, and NoF. More concrete words, positively valenced words, and words with a higher NoF had faster RTs. There was no significant relationship between RT and arousal, SND, and SD.

Turning to non-linear effects, the quadratic valence term accounted for an additional 0.7% of variance, *F*_change(1, 344)_ = 5.31, *p* = 0.022. Like the LDT, the relationship between valence and RTs was represented by an inverted U, with strongly positive and negative words eliciting faster RTs than neutral words. Arousal did not interact with either linear or quadratic valence, *F*_change_ = 1.26, *p* = 0.285.

In addition to the item-level regression analyses, we also analyzed the data using a linear mixed effects (LME) model to determine if the effects of semantic richness variables were moderated by task. Using R (R Core Team, 2015), we fitted reciprocally transformed RT data (–1/RT) from both tasks (Masson and Kleigl, 2013), using the lme4 package (Bates et al., 2015); *p*-values for fixed effects were obtained using the lmerTest package (Kuznetsova et al., 2016).

The influence of lexical and semantic richness variables, as well as the task by variable interactions, were treated as fixed effects. Effect coding was used for the dichotomous task variable, whereby lexical decision was coded as –0.5 and semantic categorization as 0.5. Random intercepts for participants and items, and random slopes for frequency, number of features, concreteness, and valence were also included in the model. As can be seen in Table 5, the pattern of effects for the lexical and semantic richness variables converge with the results obtained in the item-level regression analyses. Specifically, with respect to the semantic richness dimensions, the effects of concreteness, NoF, and valence (linear and quadratic) were reliable, but not arousal, SND, and SD. There was a significant interaction between number of morphemes and task, in which the inhibitory influence of number of morphemes was stronger in the LDT; this is consistent with a greater emphasis on lexical-level processing in lexical decision. Interestingly, there was also a significant concreteness × task interaction, wherein the facilitatory influence of concreteness was stronger in the SCT. This finding will be considered further in the Discussion.

**Table 5:** **Linear mixed model estimates for fixed and random effects**.

**Random effects**	**Variance**	***SD***	
**ITEMS**			
Intercept	0.0063	0.0793	
**PARTICIPANTS**			
Intercept	0.0093	0.0963	
Frequency	0.0004	0.0193	
Structural PC	0.0001	0.0082	
Concreteness	0.0001	0.0091	
**Random effects**	**Coefficient**	**Standard error**	***p*****-value**
Intercept	−0.7930	0.1640	<0.001
Task	0.4131	0.0989	<0.001
Word duration	0.0007	0.0000	<0.001
Log subtitle word frequency	−0.0479	0.0128	<0.001
Uniqueness point	0.0058	0.0048	NS
Structural principal component	−0.0526	0.0068	<0.001
No. of morphemes	0.0235	0.0116	<0.05
Concreteness	−0.1270	0.0336	<0.001
Valence	−0.0239	0.0055	<0.001
Quadratic valence	−0.0105	0.0038	<0.01
Arousal	0.0050	0.0057	NS
Number of features	−0.0033	0.0015	<0.05
Semantic neighborhood density	−0.0004	0.0686	NS
Semantic diversity	−0.0061	0.0206	NS
Log subtitle word frequency × Task	0.0098	0.0085	NS
Uniqueness point × Task	0.0014	0.0028	NS
Structural principal component × Task	0.0070	0.0040	NS
No. of morphemes × Task	−0.0131	0.0066	<0.05
Concreteness × Task	−0.0741	0.0197	<0.001
Valence × Task	−0.0004	0.0032	NS
Quadratic valence × Task	0.0020	0.0022	NS
Arousal × Task	−0.0037	0.0033	NS
Number of features × Task	−0.0015	0.0008	NS
Semantic neighborhood density × Task	0.0692	0.0400	NS
Semantic diversity × Task	−0.0064	0.0120	NS

## Discussion

The goal of the present study was to determine the unique contribution of semantic richness variables, above and beyond the contribution of lexical variables, to spoken word recognition in lexical decision and semantic categorization tasks. Similar relationships between the lexical control variables and latencies were found across both tasks, and the direction of the findings were congruent with past research. Word frequency effects, where common words were responded to faster, were manifested in the significant negative relationship between RTs and frequency. The robust effects of lexical competition in the form of increased word-form similarity slowing spoken word recognition were replicated in the negative correlation between RT and the structural PC. Words with more similar sounding or closer neighbors were associated with slower recognition speed. In both tasks, words whose tokens had longer durations took longer to recognize, while in lexical decision, words with more morphemes took longer to classify as words.

### Semantic richness effects in spoken word recognition

Turning to the semantic richness effects, several findings were consistent with some of the visual word recognition literature. First, semantic richness effects collectively accounted for more of the unique variance in explaining RTs in the SCT (7.5%) than in the LDT (3.0%), after controlling for the variance explained by lexical variables, consistent with Pexman et al. (2008). Second, the more concrete the word, the faster the response (see Schwanenflugel, 1991); which also corroborates Tyler et al.'s (2000) findings in auditory LDT. Third, there was evidence for both a linear and quadratic effect of emotional valence. That is, positive words generally elicited faster response times, but there was also an inverted U-shaped trend, which was reflected by faster latencies for very positive and very negative words, compared to neutral words. In other words, our data are consistent with studies that have reported linear (Kuperman et al., 2014) *and* non-linear (Kousta et al., 2009) effects. We also found no evidence that valence effects (either linear or non-linear) were moderated by arousal, consistent with Estes and Adelman (2008) and Kuperman et al. (2014); this suggests that valence effects generalize across different levels of arousal. Fourth, high NoF words were associated with faster RTs (see Pexman et al., 2003, 2008), which also corroborates Sajin and Connine's (2014) findings in auditory LDT.

These findings suggest that semantics do contribute to spoken word recognition. Concreteness and NoF influences could be accommodated by processing mechanisms that include bi-directional feedback between semantic and lexical/phonological representations (Pexman, 2012). Words that are more concrete and have more features are presumably receiving more feedback activation from the semantic feature units and will cross the recognition threshold faster. Interactive activation models of speech perception such as TRACE (McClelland and Elman, 1986), the Distributed Cohort Model (Gaskell and Marslen-Wilson, 1997), and the domain-general interactive activation and competition framework by Chen and Mirman (2012) are well placed to accommodate semantic influences because the architecture accommodates feedback mechanisms. Models that assume a modular architecture (e.g., Forster, 1979) or are fully thresholded such as Merge (Norris et al., 2000) do not incorporate feedback mechanisms from higher levels. It would be less straightforward for these models to explain semantic influences as it would mean that responses for the lexical and semantic tasks would have to be based on the semantic level rather than lexical or structural levels. The Neighborhood Activation Model (Luce and Pisoni, 1998) may be able to accommodate semantic effects as it allows for higher order effects such as a frequency bias, but at present semantic influences remain unspecified.

### Comparing richness effects across modalities

Three findings of the present study are only partly consistent with the visual word recognition literature. First, previous work found SND effects in LDT but not SCT (see Pexman et al., 2008; Yap et al., 2011), whereas the present study did not find SND influences for both tasks. Second, there was no effect of SD in either task in the present study, whereas in visual word recognition, word ambiguity generally facilitates lexical decision RTs but has no effect on semantic classification RTs (see Yap et al., 2012). Third, there was no relationship between arousal and RTs in the present study, but more highly arousing words have been found to slow RTs in visual word recognition (Kuperman et al., 2014). However, it should also be noted that the arousal effect reported by Kuperman and colleagues, despite being statistically significant, accounted for very little variance (0.1%) in LDT RTs. Indeed, in carefully controlled factorial experiments, it has been difficult to detect arousal effects in lexical processing (e.g., Kousta et al., 2009). We also note that the differences found between the present study on spoken word recognition and previous studies on visual word recognition could be due to the fact that most of the values and ratings for the semantic richness variables were based on written words rather than spoken words; this will require future research to investigate.

The pattern of results suggests that the influence of concreteness and NoF in word recognition generalizes consistently across the visual and spoken modalities. It appears that these two dimensions generalize widely across tasks in both modalities—Yap et al. (2012) observed that concreteness (imageability) and NoF effects were found in all five tasks in their study, whereas effects such as SND and SD are less stable. Also consistent across modalities is the finding that semantic richness effects are more evident in SCT than LDT. We also found a task × concreteness interaction in the LME analysis, in which the facilitatory effect of concreteness was larger in the SCT than in the LDT. The SCT requires participants to discriminate between concrete and abstract words, and the concreteness ratings of for concrete words are, by definition, higher than those for abstract words. This encourages participants to rely on the concreteness dimension to drive the concrete/abstract binary decision, thereby exaggerating the size of concreteness effects. This is consistent with Yap et al.'s (2012) observation of larger effects of imageability in semantic categorization, compared to lexical decision.

The apparent lack of influence for some semantic dimensions such as SND and SD may indicate that the degree of semantic influence in spoken word recognition may be smaller than in visual word recognition. If we examine the amount of variance explained in the regression analyses for this study and the one in Pexman et al. (2008), which also looked at lexical and semantic contributions to LDT and SCT for printed words, it can be seen that they are quite comparable in LDT: it was 44% for lexical variables and a 4% increase for the unique variance in RT explained by semantic richness for both studies. However, for SCT, it was 10% for lexical variables in Pexman et al. vs. 37% in the present study, with a 10% increase in semantic richness in Pexman et al. vs. a 7.5% increase in the present study. In auditory SCT, it appears that the contribution of semantic factors relative to lexical factors is actually much smaller—far more variance is accounted for by lexical factors compared to Pexman et al. We have argued elsewhere (see Goh et al., 2009) that some of the potentially late occurring processes in spoken word recognition, such as the frequency bias in the word frequency effect, may be secondary to the more fundamental problem of resolving acoustic-phonetic identity arising from form-based competition among similar sounding word candidates. As discussed in the Introduction, the differences in the nature of the interaction between neighborhood density and word frequency in spoken versus visual word recognition may be attributed to the more pressing need to resolve phonological similarity competition in the spoken domain. The smaller richness effects in spoken word recognition may again reflect the primacy of form-based competition in this modality.

Nevertheless, semantic richness does play a role and should be investigated more thoroughly in the field of spoken word recognition to further advance our knowledge of the underlying mechanisms that allow us to understand what others are saying. The relative contributions of the different semantic dimensions to auditory LDT and SCT add to the previous factorial studies that have examined semantic variables one at a time. The present findings indicate that concreteness, NoF, and valence influence spoken word recognition across both LDT and SCT, and in the same direction, which supports the task-generality of these semantic richness effects. That being said, it is clear that task-specific effects are also apparent in the auditory modality. Specifically, as mentioned earlier, Yap et al.'s (2012) observation of stronger imageability effects of semantic categorization, relative to lexical decision, was mirrored in our finding that concreteness effects are exaggerated in a binary decision task that places a premium on concreteness as a discriminating dimension. Effects of SND, SD, and arousal were not evident in both tasks. It remains to be seen if these semantic richness effects, or lack thereof, can be generalized across more tasks. Future studies should look into other tasks, word sets, and languages in order to afford a better understanding of how meaning influences our ability to recognize words in spoken language.

### Future directions and concluding remarks

The results of the present study extend the semantic richness literature by demonstrating that to a large extent, the key findings in the visual modality are for the most part generalizable to the spoken modality, although there are some theoretically interesting differences. Since we wanted to make our study as comparable as possible to Pexman et al.'s (2008) seminal study, we used their stimuli (i.e., the concrete words in McRae et al.'s, 2005, feature-listing norms) and paradigms. This means that our analyses are necessarily limited to concrete nouns, and future research can explore semantic influences on the processing of spoken abstract words. Indeed, the semantic representation of abstract concepts remain poorly understood, even in the visual modality (Pexman et al., 2016). Another limitation was that we did not collect gender and age information of the participants, which could constrain comparisons with other samples.

From a more methodological perspective, there is evidence that task parameters can shape the magnitude and direction of empirical effects. For example, as discussed earlier, Pexman et al. (2003) showed how the specific decision (e.g., abstract/concrete vs. living/nonliving) chosen for a semantic task can affect the magnitude of NoF effects. Future research can explore the nature of semantic richness effects in spoken word recognition when other types of semantic decisions are required of the participant. Related to this, it is well-established that the wordlikeness of *nonword* distracters in lexical decision can moderate the effect of different lexical properties on word processing (e.g., Stone and Van Orden, 1993; Carreiras et al., 1997). In the visual word recognition literature, nonword wordlikeness can be manipulated by using pseudohomophones (i.e., nonwords that sound like real words, e.g., *brane*) or unpronounceable nonwords (e.g., *brata*). Practical constraints preclude the use of pseudohomophones or unpronounceable nonwords in auditory lexical decision, but one could manipulate wordlikeness by selecting nonwords that vary on phonological neighborhood density. It will be interesting to investigate the extent to which semantic richness effects vary as a function of nonword type in auditory lexical decision.

In summary, the present findings help to further constrain our understanding of semantic processing in spoken word recognition. Our results add to a growing literature establishing that semantic representations are multidimensional, dynamic, and context-sensitive (Pexman et al., 2013).

## Author contributions

WG and MY conceptualized and designed the study. ML, MN, and LT created the materials, collected and processed the data. All authors were involved in the data analyses and interpretation. WG drafted the report and all authors were involved in the revision.

### Conflict of interest statement

The authors declare that the research was conducted in the absence of any commercial or financial relationships that could be construed as a potential conflict of interest.
